# Harnessing Biofilm-Mediated Plastic Biodegradation: Innovating Smart Material Design

**DOI:** 10.1021/acsaenm.5c00179

**Published:** 2025-07-02

**Authors:** Kaitlyn Benes, Madison Liguori, Cody J. Velikaneye, Sarah Kispert, Alexis Pishnyuk, Eddie Luzik, Hao Sun, Dequan Xiao, Huan Gu

**Affiliations:** Department of Chemistry & Chemical Engineering and Biomedical Engineering, Tagliatela College of Engineering, 8518 University of New Haven, West Haven, Connecticut 06516, United States

**Keywords:** synthetic plastic, petropolymers, biofilm, biodegradation, enzymes, enzyme kinetics

## Abstract

The persistent environmental challenges posed by synthetic plastics, particularly petroleum-derived petropolymers, such as polyethylene (PE), polypropylene (PP), and polystyrene (PS), have intensified the need for innovative recycling methods. Traditional recycling techniques often rely on harsh conditions, raising environmental and economic concerns. Biofilm-mediated biodegradation has emerged as a promising alternative, operating under mild conditions such as room temperature, neutral pH, and atmospheric pressure. However, the interactions between biofilm-forming microorganisms and synthetic plastics and the roles of secreted enzymes in these processes remain incompletely understood. This review explores the current understanding of biofilm-mediated biodegradation—biodeterioration, biofragmentation, bioassimilation, and mineralization—and the biochemical and physical interactions that control these processes. We highlight the latest findings on the enhancement of petropolymer degradation by biofilms, focusing on the roles of oxidative and attachment enzymes and the environmental factors influencing degradation efficiency. Understanding these complex interactions can inform the design of next-generation enzyme-responsive polymers that are not only easier to degrade but can also serve as smart materials for diverse applications, such as antifouling coatings on metals. This perspective bridges critical knowledge gaps and provides insights into harnessing biofilm-mediated processes for sustainable material innovation.

## Graphical abstract

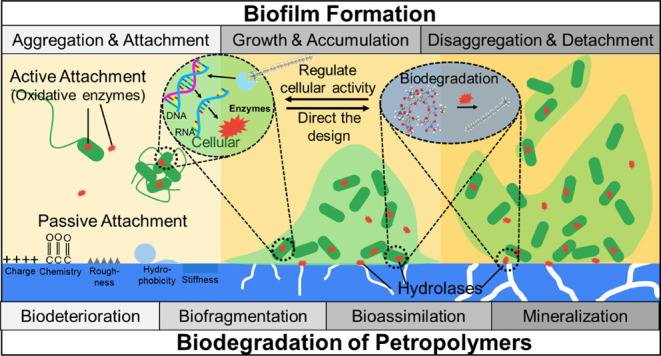

## 1 Introduction

### 1.1 Background on Petroleum-Derived Polymers

Petroleum-derived polymers, commonly known as petropolymers, form the backbone of modern materials and serve essential roles in consumer products, industrial applications, and medical devices. Among them, thermoplastics such as polyolefins [polyethylene (PE), polypropylene (PP), and polystyrene (PS)], polyvinyl chloride (PVC), and polyacrylates are widely utilized due to their durability, flexibility, and cost-effectiveness.^1,2^ In addition, condensation polymers like polyethylene terephthalate (PET) and the thermoset polyurethane (PU) significantly contribute to packaging, textiles, and structural materials.^1,2^ These hydrophobic polymers are synthesized through the polymerization of small-molecule olefins (ethylene and propylene, respectively), mainly derived from low-molecular-weight constituents of petroleum or natural gas fractions.^2^ Their widespread use, however, has led to an escalating environmental crisis as the accumulation of plastic waste presents serious ecological hazards. This challenge is exacerbated by the hydrophobicity and chemical inertness of petropolymers, which severely hinder natural degradation pathways.^3,4^

### 1.2 Background on the Degradation Strategies of Petroleum-Derived Polymers

Conventional recycling strategies, including mechanical and chemical recycling, aim to mitigate plastic pollution by repurposing or breaking down polymers into reusable materials.^5–8^ Mechanical recycling involves the collection, sorting, and reprocessing of plastics through shredding and remelting, often resulting in secondary products of lower quality due to polymer chain degradation and contamination.^8^ Chemical recycling, particularly depolymerization, offers a more theoretically robust solution by converting polymers into their constituent monomers for subsequent repolymerization, thereby yielding recycled petropolymers that match or surpass the original materials’ physicochemical attributes.^5–7^ Chemical treatment and thermal pyrolysis have shown some promise in facilitating petropolymer degradation; however, these approaches often require harsh reaction conditions (e.g., high temperatures exceeding 500 °C) and toxic organic solvents, raising concerns about environmental and energy implications.^3^ Moreover, hydrophobic petropolymers (e.g., PE, PP, and PS) are extremely recalcitrant to depolymerization due to the low polarity of C–C and C–H bonds, which have only a few poorly selective cleavage reactions as compared to the C–O and C–N bonds found in other polymers such as polyethylene terephthalate (PET).^3^ This resilience is further compounded by the diversity within the PE family itself, including high-density polyethylene (HDPE), low-density polyethylene (LDPE), and linear low-density polyethylene (LLDPE), with HDPE presenting the greatest challenge due to its higher density and crystallinity.

Consequently, in recent years, biodegradation is attracting ubiquitous attention because it is a mild and eco-friendly method; however, its practical application remains limited by the slow degradation rates of most petropolymers and the low enzymatic specificity toward hydrophobic backbones.^3,9^ The search for more efficient biorecycling strategies, particularly those leveraging biofilms and microbial consortia, has gained significant interest as a means of overcoming these limitations. In this review, we aim to summarize the current status of biodegradation, identify the opportunities for using biofilms to enhance biodegradation, and discuss how biodegradation can direct the design of next-generation eco-friendly polymers. While previous reviews have focused on the biofilm-mediated biodegradation of microplastics in aquatic environments,^10^ this review will specifically address the degradation of bulk petropolymer films and pieces.

## 2 Review of the Efficiency of Biofilm-Mediated Biodegradation

Microorganisms such as bacteria, fungi, or algae are ubiquitous in natural environments such as soil, oceans, and wastewater systems.^10^ These microorganisms possess a remarkable ability to adhere to solid surfaces and construct “houses,” matrix-embedded communities known as biofilms, using self-produced extracellular polymeric substrates (EPS).^10–12^ Biofilms provide microorganisms with a structured and resilient microenvironment that enhances their survival against harsh and fluctuating external conditions, such as extreme temperatures, desiccation, and antimicrobial agents.^13^ This capacity not only allows microorganisms to adhere to the solid surfaces but also enables them to continuously modify these surfaces through their metabolic activities (e.g., electron scavenging) and secreted products (e.g., enzymes and corrosive compounds). As a result, biofilm-forming bacteria act as living biocatalysts with significant potential for biodegradation.^10,14–16^ Within biofilms, microbial consortia operate synergistically to degrade complex substrates, including synthetic polymers. Typically, one group of microorganisms initiates plastic depolymerization by secreting oxidative and hydrolytic enzymes, such as esterases, cutinases, and laccases, which break down macromolecular plastic structures into oligomers and monomers. Subsequently, this group and/or secondary microbial populations metabolize these intermediates, converting them into smaller, bioassimilable molecules, such as organic acids and CO_2_.^17,18^ Notably, biofilms exhibit persistence, stability, self-generation, and self-healing capabilities, making them exceptionally well-suited for biodegradation strategies.^19^

Several microorganisms have demonstrated enhanced plastic degradation capabilities through biofilm formation (Figure 1 and Table S1). For instance, biofilms formed by cold plasma-pretreated BS-10L cells on LDPE films caused a 27.78 ± 0.014% mass loss compared to 13.4 ± 0.013% by the untreated BS-10L cells after 30 days of incubation at 30 °C in minimal salt medium (MSM) due to the formation of increased cracks, holes, and roughness on the surface of LDPE films (Figure 1a).^20^ Cold plasma treatment was used to increase surface energy and enhance biofilm formation.^20^ Microbial colonization by on LDPE films caused a 33.75% weight loss after 21 days of incubation at 30 °C with shaking (Figure 1b).^21^ biofilms have been shown to accelerate PET degradation by producing PETase and MHETase enzymes (Figure 1b).^21^ Likewise, and species exhibit robust biofilm-mediated degradation of polyolefins and polyurethane due to their ability to secrete oxidative enzymes and biosurfactants that enhance polymer accessibility (Figure 1b).^21^ C208 biofilms can degrade PE at a rate of 0.86% per week in 100 mL nutrient broth at 30 °C with shaking (Figure 1c).^22^ Protease promotes the formation of more robust and multilayered C208 biofilms for biodegradation compared to trypsin (Figure 1d).^14^ Bacterial strains *Comamonas*, *Delftia*, and *Stenotrophomonas* have been shown to degrade PE, reducing the material by 46.7% of its viscous area after 90-day incubation (Figure 1e).^23^

**Figure 1:**
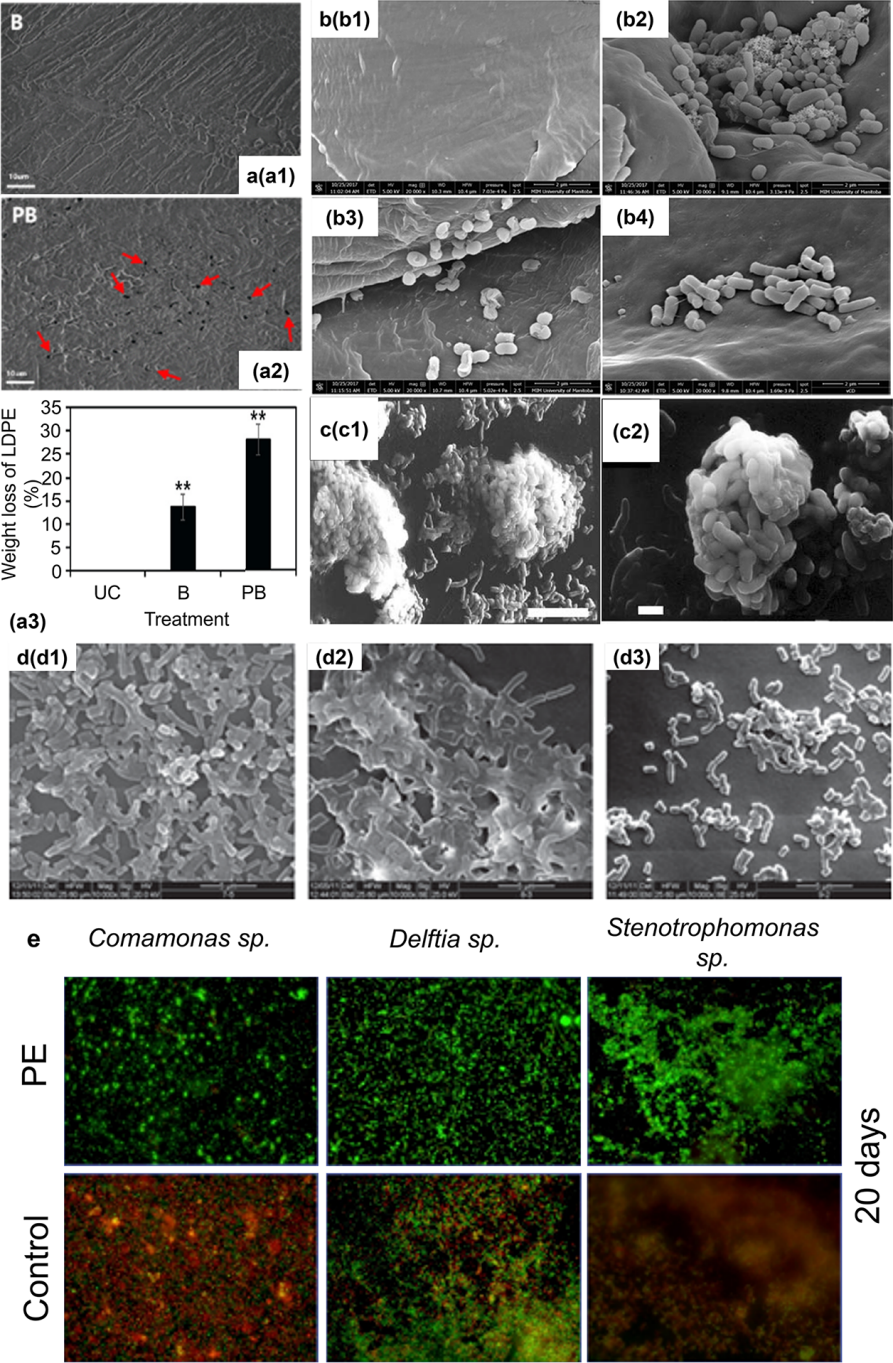
Efficiency of biofilm-mediated biodegradation. (a) Degradation of LDPE films and particles by BS-10L. (a1,a2) Confocal images of untreated (B) and plasma-activated (PB) BS-10L biofilms on LDPE films after 60 days of incubation at 30 °C. Red arrows indicate visible surface holes caused by biofilm-mediated degradation. PB shows more dense and adherent biofilm structure. (a3) Quantified dry weight loss of LDPE particles incubated with untreated control (UC), untreated BS-10L (B), or plasma-treated BS-10L (PB).^20^ Reproduced with permission from ref. 20. Copyright 2025 Elsevier. (b) SEM images of LDPE surfaces incubated at 30 °C for 300 h: (b1) no biofilm, (b2) LS46, (b3) H16, (b4) IRN19. and formed more uniformly distributed and embedded biofilms with distinct surface roughening.^21^ Reproduced with permission from ref. 21. Copyright 2025 Canadian Science Publishing. (c) SEM of C208 biofilm after 20 h incubation on PE film at 30 °C.^22^ The biofilm appears patchy with micropits. Reproduced with permission from ref. 22. Copyright 2025 Springer Nature. (d) SEM images of C208 on polystyrene wells: (d1) untreated, (d2) treated with proteinase, (d3) treated with trypsin, showing varying EPS density.^14^ Reproduced with permission from ref. 14. Copyright 2025 Oxford Academic. (e) Confocal images of biofilms formed by *Comamonas* sp., *Delftia* sp., and *Stenotrophomonas* sp. on PE sheets after 20 days. Biofilm structure differs in thickness and spatial distribution across species.^23^ Reproduced with permission from ref. 23. Copyright 2025 Elsevier.

## 3 Review of Key Factors Influencing Biofilm-Mediated Degradation

Currently, it is widely recognized that biodegradation begins with biofilm formation.^24,25^ The process of biofilm development on petropolymer films in the environment follows three stages—aggregation and attachment, growth and accumulation, and disaggregation and detachment (Figure 2).^26^ These biological stages align closely with the four key stages of biodegradation: biodeterioration, biofragmentation, bioassimilation, and mineralization.^3,17^ More importantly, biodegradation is initiated as early as the aggregation and attachment stages, progressing in tandem with biofilm formation. Each stage of biofilm development influences the effectiveness and progression of biodegradation through critical controlling factors.

**Figure 2:**
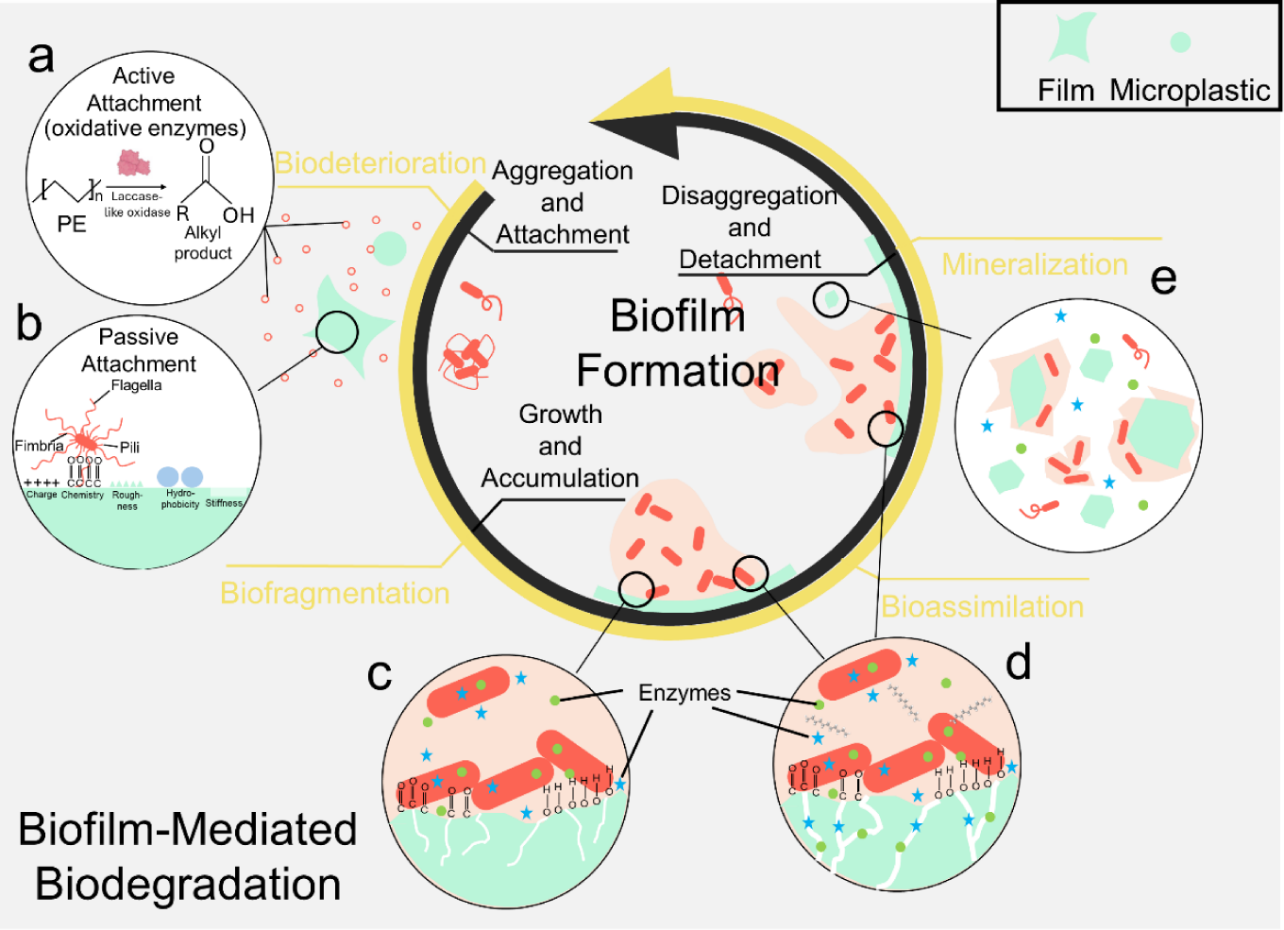
Alignment of biofilm formation steps with biofilm-mediated biodegradation of petropolymers. The biofilm formation steps include aggregation and attachment, growth and accumulation, and disaggregation and detachment. The biofilm-mediated biodegradation steps include biodeterioration during active (a) and passive (b) attachment of biofilm formation, biofragmentation (c), bioassimilation (d), and mineralization (e).

### 3.1 Review of Key Factors Influencing Biodegradation during the First Step of Biofilm Formation: Aggregation and Attachment (Figure 2a,b)

Aggregation and attachment are the first steps of biofilm formation.^26^ This process can occur through individual free-swimming bacteria or bacterial aggregates in solution.^26^ Microbial attachment onto petropolymer films can be categorized as either active or passive.^10^ In active attachment, microorganisms directly utilize petropolymer surfaces as carbon sources rather than relying on flagella and pili for movement, as seen in traditional attachment (Figure 2a). Consequently, the first step of biodegradation, biodeterioration (Figure 2a), begins during the active attachment. Free-swimming microorganisms release oxidative enzymes that facilitate carbon utilization from petropolymer surfaces by reacting with available oxygen, nitrate, or ferric ions in the aqueous environment (Table 1). For instance, secretes laccases that oxidize PE surface chains, introducing carbonyl and hydroxyl functional groups and enhancing hydrophilicity, which promotes microbial colonization and initiates biodeterioration.^27^ Similarly, releases PETase, an oxidative-hydrolytic enzyme that specifically cleaves the ester bonds in polyethylene terephthalate, producing mono­(2-hydroxyethyl) terephthalate, which is then further metabolized, advancing the biodegradation process.^28^ These reactions introduce functional groups such as C=O,^10^ O–H, and amine onto the polymer chains or at cleavage sites.^29–31^ The activities of oxidative enzymes can also promote bacterial adhesion by increasing surface roughness (e.g., creating pits, cracks, and other surface irregularities)^32,33^ or by altering the surface properties of petropolymers from hydrophobic to hydrophilic,^3,17^ thereby enhancing microbial colonization.

**Table 1:** Summary of the Oxidative Enzymes and Their Roles in Biofilm Formation During Biodegradation

Enzymes	Effects on biofilm formation: (+): enhancing/promoting (−): inhibiting/decreasing(n): either (+) or (−) depending on the circumstances	Microorganisms	Petro-polymers	Ref
Laccase	(+): increasing hydrophobicity due to the formation of C=O and O–H bond (Figure 2c,d) and providing extra area for bacterial adhesion. (n): producing medium-chain-length alcohols and ketones.	C208	PE	^22,31,44,45^
			PU	^33^
Manganese peroxidase	(+): formation of C–O bond (Figure 2c,d).		PET	^46^
Soybean peroxidase (*Soybean*)	(+): increasing hydrophobicity due to the increase of O/C atomic ratio of HDPE surfaces and formation of −CO– and surface became rougher.	*Soybean*		^47^
Cytochrome P450	(+): increasing hydrophobicity due to the formation of C=O, O–H, and amide bond (Figure 2c,d).	JNU01		^30,44^
PETase	(+): increasing surface roughness by creating pits, cracks, and other surface irregularities as polymer is broken down; increasing surface hydrophobicity; enhancing EPS production by providing a steady supply of nutrients.	201-F6		^48^
Dioxygenase (HIS1)		*Prediction by the programmed sequence-based oxygenase screening*	PP	^49^
Hydroquinone peroxidase	(+): change in surface chemistry, increase in hydrophobicity, and increase in surface roughness.	HM121	PS	^50,51^
Alkane hydroxylase	(+): overexpression of AlkB enzyme could alter the hydrophobic interactions between bacteria and hydrophobic surfaces due to the production of hydrophilic alcohols from alkanes. (n): overexpression of AlkB enzyme could promote biofilm formation if alkanes are available.	*Pseudomonas* sp. E4 E7 (uniport Q9I0R2)	PE	^47,52^
		JNU01	PS	^74^

Moreover, oxidative enzymes for biodeterioration and biofilm attachment proteins are interconnected through their roles in biofilm dynamics.^34,35^ Understanding and harnessing these interactions offer valuable insights into microbial behavior and potential strategies for biofilm management, controlling biofilm-mediated biodegradation, and directing the future design of biodegradable petropolymers. For instance, *in vitro*, the production of the carbon oxidative enzyme glucose oxidase, which converts glucose to gluconic acid and hydrogen peroxide, can inhibit biofilm formation of , methicillin-resistant , and .^36^ In , the operon regulating lactate oxidase, an oxidative enzyme that converts lactate to pyruvate and hydrogen peroxide, and the operon for biofilm formation (LutR) are coregulated by the same GntR-type repressor.^37^ This dual regulation links oxidative metabolism to surface colonization, suggesting a coordinated physiological response that could be advantageous for polymer degradation. From a synthetic biology perspective, regulatory elements such as GntR-type repressors could be engineered into microbial platforms to synchronize enzyme secretion and biofilm formation in response to plastic-derived intermediates. This would enable targeted activation of biodegradation pathways under environmentally relevant conditions, improving the system efficiency and specificity. In , the regulator of pyruvate oxidase that converts pyruvate to acetyl-CoA and carbon dioxide during biodeterioration, also controls DNA release and bacterial adhesion.^38^ Moreover, oxidative enzymes can produce reactive oxygen species (ROS) that might oxidize or modify attachment proteins such as surface proteins,^39^ pili/fimbriae,^40^ or polysaccharide intercellular adhesin (PIA),^41^ potentially affecting their binding ability during attachment.^42^

Beyond aggregation and attachment, oxidative enzymes like glucose oxidase and lactate oxidase can subsequently influence oxidative stress within biofilms, impacting the stability and integrity of the biofilm matrix and its attachment properties.^36,37,42^ The reactive products from oxidative enzymes might interact with or disrupt biofilm matrix components, potentially affecting bacterial attachment and biofilm resilience.^42^ In some cases, oxidative enzymes might be utilized to disrupt biofilms by affecting the matrix or attachment proteins, offering potential strategies for biofilm control.^36,42^ Bacterial attachment proteins are a class of surface-associated proteins and secreted enzymes (e.g., sortases, autotransporter adhesins, and pili-associated glycosyltransferases) that assist microbial cells in adhering to solid substrates and forming biofilms by anchoring surface proteins or pili to bacterial cell walls and facilitating stable contact with polymer surfaces.^43^ For example, TasA in forms amyloid fibers that stabilize the biofilm matrix, while LapA in acts as a large adhesin that anchors cells to hydrophobic polymer surfaces. Additionally, interface-active enzymes, such as esterases and lipases, can modify the polymer surface chemistry, enhancing both attachment and initiating degradation. These mechanisms play a critical role in the early stages of biofilm-mediated biodegradation by coupling physical adhesion with enzymatic action. Understanding how carbon oxidative enzymes interact with bacterial attachment enzymes is crucial for manipulating biofilm adhesion, offering a basis for further exploration of these mechanisms and their potential applications in controlling biofilms.

In contrary to active attachment, passive attachment means that microorganisms attach to petropolymer surfaces via biochemical and physical interactions.^10^ This refers to interactions occurring during attachment rather than microbial movement toward the surface due to gravity (Figure 2b). Recent reviews have summarized the roles of biochemical and physical interactions in microbial attachment.^53–55^ Therefore, this review specifically focuses on how these interactions influence biomaterial attachment to petropolymer films. From a biochemical perspective, petropolymer chains (e.g., PE, PP, or PS) vary in functional groups and 3D molecular structures (Figure 3), affecting their crystallinity, surface charge, and hydrophobicity—factors known to influence microbial adhesion. Petropolymer surfaces are typically hydrophobic and exhibit low charge and surface energy due to their nonpolar hydrocarbon chains. However, environmental exposure modifies these surfaces through chemical and microbial interactions. Microorganisms are generally negatively charged due to peptidoglycan in their cell walls or lipopolysaccharides (LPS) and phospholipids in their membranes.^56^ As a result, they preferentially attach to positively charged surfaces and are repelled by surfaces with a weak or negative net charge, such as petropolymers.^57^ Hydrophobic microbes exhibit stronger adhesion to these materials and can actively adjust their surface hydrophobicity under carbon starvation to enhance attachment.^58,59^ To promote biofilm formation for enhanced biofilm-mediated biodegradation, new biodegradable petropolymer surfaces could be engineered with positively charged functional groups or increased hydrophilicity. Treatments such as plasma activation, chemical etching, or coatings that introduce polar functional groups can also be used to enhance microbial adhesion by making PE, PP, or PS surfaces more hydrophilic. It is important to note that this strategy is intended for biodegradable petropolymers designed for postuse degradation under controlled conditions, such as industrial composting or biofilm-enabled treatment systems. In this context, promoting microbial adhesion is beneficial, as it facilitates more efficient enzymatic breakdown, unlike conventional polymers where biofilm formation is typically undesirable.

**Figure 3:**
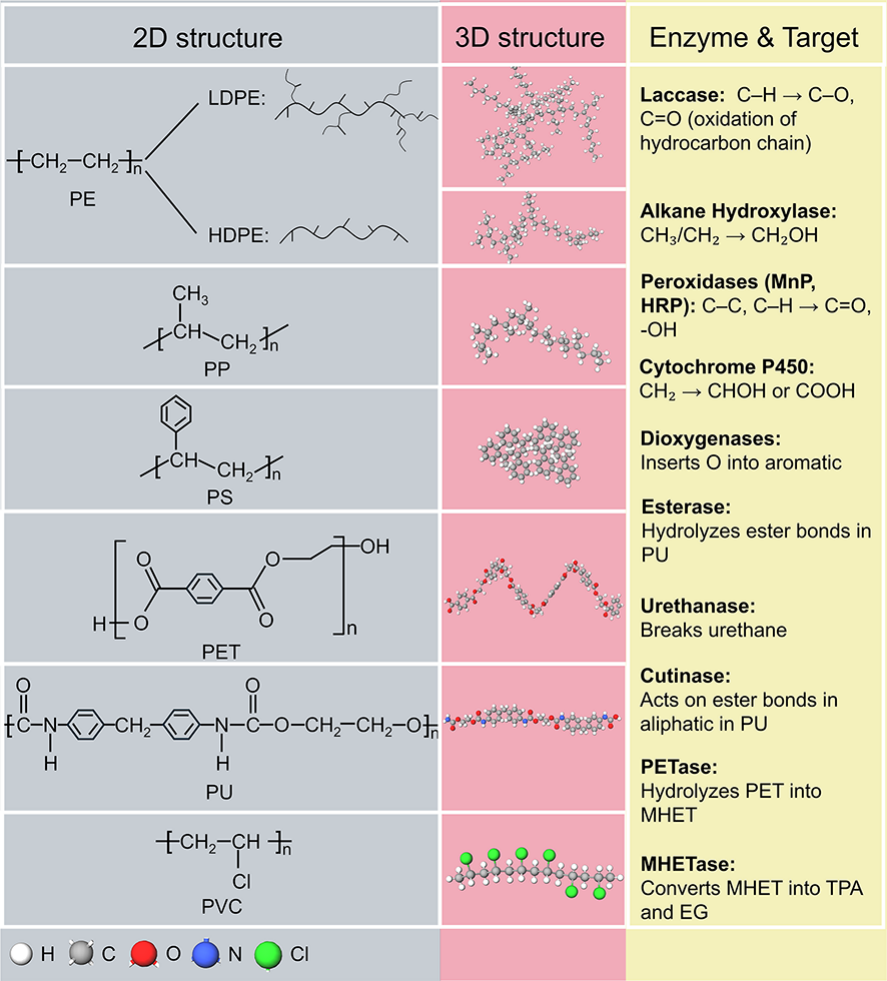
Summary of petropolymers’ 2D and 3D structures and the examples of enzymes with their target functional groups in petropolymers.

Physical interactions during passive microbial attachment can be influenced by the characteristics of the surface, or surface texture that includes roughness and 3D configuration.^53,57^ Petropolymer films in the environment are not fully smooth.^60^ Surface texture can affect microbial adhesion by different mechanisms. First, surface texture could affect the wettability of a surface.^61^ Second, when the dimension of surface texture is smaller than the body length of a microorganism, it can inhibit bacterial adhesion because cell–cell interactions are essential to bacterial adhesion.^62^ When the dimension of surface texture is larger than the microorganisms’ body length, surface texture offers more area for microbial adhesion.^62^ Lastly, in nature, microbial adhesion onto petropolymer films could be accomplished under various flow conditions (e.g., lake, ocean with tides, or river). The movement of fluids over a surface can impact biofilm formation.^63^ For example, increased fluid flow toward or parallel to a substratum helps microbial adhesion due to higher mass transport, despite the presence of higher fluid shear stimulating their detachment.^64^ However, when fluid flow exceeds a critical limit, resulting wall shear rates may become high enough to prevent adhesion or even stimulate detachment.^65^ It is worth noting that adhesive forces vary among strains and species. For example, the adhesion forces of pili are 95 pN^66^ and the lateral detachment forces of cells on polished stainless steel and a glass slide coated with poly l-lysine were measured as 0.763 ± 0.167 and 0.639 ± 0.136 nN, respectively, using atomic force microscopy (AFM).^67^

Interactions (e.g., collaboration or competition) among different species in the environment can also affect the adhesion of microorganisms onto petropolymers’ surfaces. It is known that the adhesion of one bacterial species to a surface can have a negative, positive, or neutral influence on other species, depending on the species involved and the nature of the substratum.^68^ Negative influence could be due to one cell having occupied or modified the surface by secreting inhibitory macromolecules. Positive influence could be attributed to the modification of the surface by the attached cells or the cell–cell contact during adhesion. Recently, extensive research has been conducted on uncovering the microbiome for plastic biodegradation (Table S1). However, the role of interspecies interactions in microbial adhesion is still not well understood, and it deserves future investigation.

### 3.2 Review of Key Factors Influencing Biodegradation during the Second Step of Biofilm Formation: Growth and Accumulation (Figure 2c,d)

Following bacterial attachment, the subsequent steps of biodegradation—biofragmentation, bioassimilation, and mineralization—typically begin. Biofragmentation (Figure 2c) involves the enzymatic breakdown of polymer carbon chains through hydrolysis or fragmentation, leading to the release of intermediate products. This process is mediated by microbial enzymes^17^ (Figure 4 and Table S2). Bioassimilation (Figure 2d) follows, where bacteria or fungi take up and metabolize the hydrocarbon fragments generated by biofragmentation.^17^ Finally, mineralization (Figure 2e) occurs, in which hydrolysis products are transported into the microbial cell, where intracellular conversion incorporates them into biomass, releasing carbon dioxide and water as byproducts.^17^ These steps are closely tied to the metabolic activity of biofilm-associated cells, yet their direct impact on biofilm formation remains unclear. Figure 4 and Table S2 provide a summary of known petropolymer-degrading enzymes and their predicted effects on biofilm formation based on their biodegradation mechanisms.

**Figure 4:**
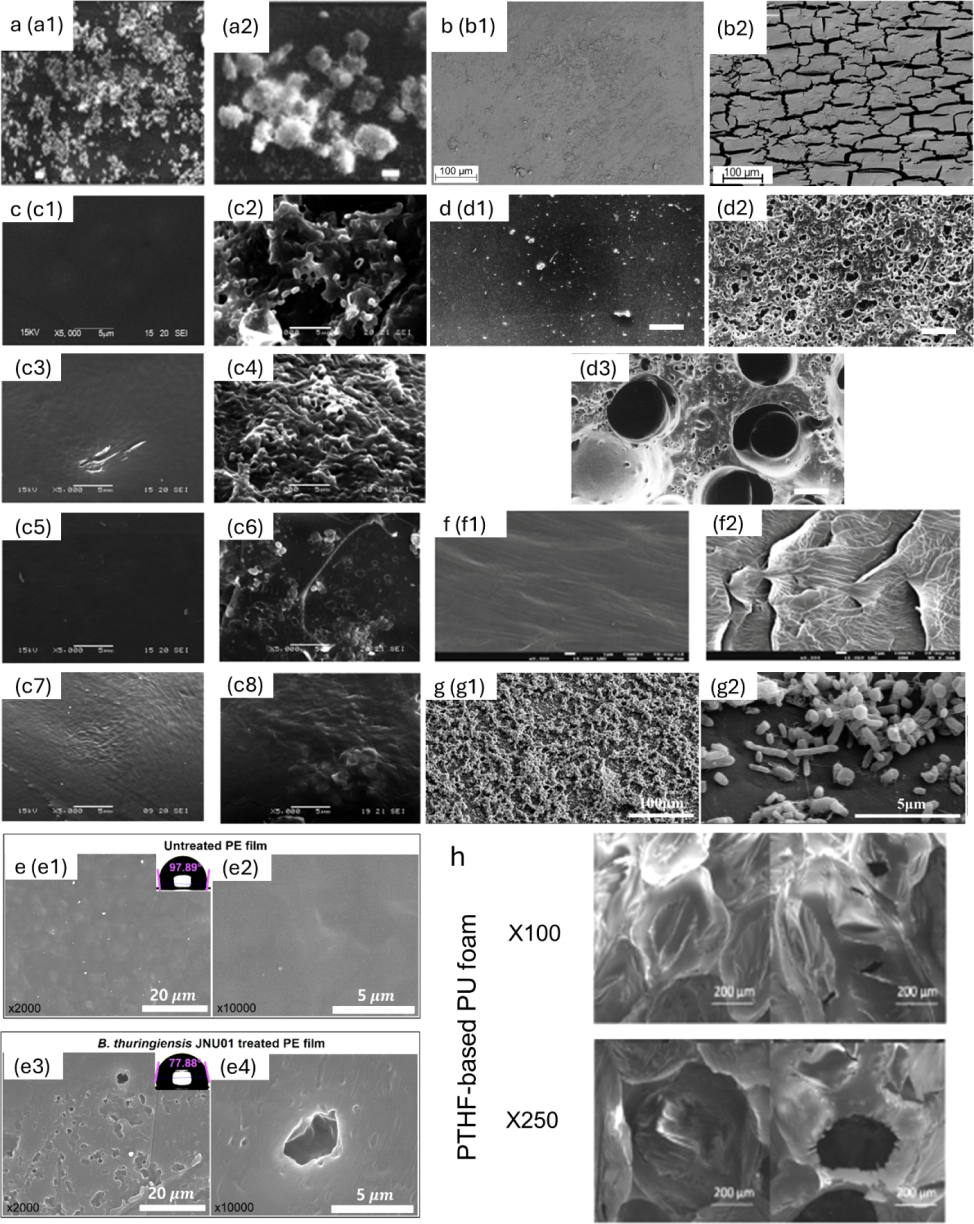
Summary of the hydrolytic enzymes for the biodegradation of petropolymers and their effects on biofilm formation during biodegradation. (a) SEM of strain C208 biofilm on polyethylene after 4 h (a2) and 20 h (a2) incubation at 30 °C (Bar = 10 μm).^22^ Reproduced with permission from ref. 22. Copyright 2025 Springer Nature. (b) SEM image of TPU surface (b1) negative control (b2) following hydrolysis by LCC at 70 °C for 200 h.^69^ Reproduced with permission from ref. 69. Copyright 2025 MDPI. (c) SEM of LMWPE sheets (PE-1, PE-2, PE-3, PE-4) before (c1, c3, c5, c7) and after (c2, c4, c6, c8) 80 days of biodegradation with *Pseudomonas* sp. at 37 °C.^52^ Reproduced with permission from ref. 52. Copyright 2025 Journal of Bioremediation & Biodegradation. (d) SEM of PUR films before (d1) and after degradation by 0.02 U (d2) and 0.2 U (d3) of PUR esterase for 24 h (Bar = 10 μm).^70^ Reproduced with permission from ref. 70. Copyright 2025 American Society of Microbiology. (e) FE-SEM of untreated PE film (e1,e2) and PE films (e3,e4) deformed by JNU01, after treatment for 1 month.^30^ Reproduced with permission from ref. 30. Copyright 2025 American Chemical Society. (f) SEM of control (f1) and H-237 bacterial degraded LDPE film (f2) after 90 days of incubation.^71^ Reproduced with permission from ref. 71. Copyright 2025 Springer Nature. (g) SEM of consortium CAS6 on PET films after 7 days incubation.^32^ Reproduced with permission from ref. 32. Copyright 2025 Elsevier. (h) SEM of degradation of PTHF based PU foams.^33^ Reproduced with permission from ref. 33. Copyright 2025 Elsevier.

Biofilm cells can secrete hydrolytic enzymes to initiate, support, or enhance biodegradation and utilize biodegradation products, such as hydrocarbons, as a carbon source to support bacterial growth under specific conditions (Figure 2c,d). For example, bacterial enzymes like PETase and laccase degrade petropolymers by hydrolyzing ester bonds, breaking them down into fatty acids and alcohols.^22,31,44,48^ Laccase in strain C208 catalyzes the oxidation of PE, breaking down its hydrocarbon backbone and leading to structural degradation (Figure 4a).^22,45^ Optimal laccase activity occurred at 70 °C, though assays were conducted at 37 °C. Copper (Cu^2+^) addition up to 20 μM/day significantly enhanced extracellular laccase production.^45^ Bacterial polyester hydrolases (e.g., LC cutinase (LCC), TfCut2, Tcur1278, and Tcur0390) degrade PU by catalyzing the hydrolysis of ester bonds within the PU structure (Figure 4b).^69^ LCC demonstrated the highest efficiency at 70 °C with up to 4.9% weight loss of TPU materials over 200 h.^69^ Alkane hydroxylase (AlkB) was from *Pseudomonas* sp. E4 is a key enzyme responsible for the biodegradation of LMWPE (Figure 4c).^52^ AlkB catalyzes the first step in alkane degradation, initiating the oxidation of polyethylene-like hydrocarbons.^52^ PUR esterase, an esterase enzyme, is the key factor responsible for the degradation of PUR by TB-35 (Figure 4d).^70^ The enzyme activity is optimized at a pH of 6.5 and a temperature of 45 °C. PUR esterase acids degrade PUR via a two-step process. In step 1, the enzyme first binds to the PUR surface through a hydrophobic domain. In step 2, the enzyme hydrolyzes the ester bonds of PUR, releasing diethylene glycol and adipic acid as degradation products.^70^ In JNU01, the CYP102A5 variant (CYP102A5.v1), a cytochrome P450 monooxygenase, is the key enzyme responsible for the biodegradation of PE (Figure 4e).^30^ CYP102A5.v1 catalyzes the hydroxylation of PE, introducing oxygen-containing functional groups like hydroxyl (−OH) and carboxyl (−COOH) to the polymer structure.^30^ In marine bacteria, laccase, manganese peroxidase, esterase, and lipase are key enzymes involved in the biodegradation of low-density polyethylene (LDPE) (Figure 4f).^71^ Laccase and manganese peroxidase break down the C–C bonds in PE, increasing surface oxidation and making the plastic more susceptible to microbial degradation. Esterase and lipase cleave ester bonds, facilitating PE breakdown.^71^ Upregulated genes encoding esterases, cutinases, and hydrolases in biofilm cells facilitate the enzymatic breakdown of PET and PE (Figure 4g).^32^ For PET, hydrolysis leads to the release of terephthalic acid (TPA) and mono-(2-hydroxyethyl) terephthalate (MHET) as primary degradation products. For PE, the degradation results in significant surface erosion and the formation of novel, unidentified degradation products.^32^ Laccase from is a key enzyme responsible for the degradation of PU with 1-hydroxybenzotriazole (HBT, 0.2 mM) as a redox mediator (Figure 4h).^33^ Laccase catalyzes the oxidation of urethane bonds and ether linkages in PU, breaking down polymer chains into smaller fragments. The addition of HBT (0.2 mM) significantly increased degradation efficiency by expanding the oxidative range of laccase, allowing it to target nonphenolic substrates like PU.^33^

The metabolic byproducts of enzymatic polymer degradation—such as alcohols and organic acids—may influence biofilm composition and structure by affecting the production of extracellular polymeric substances (EPS). Additionally, as biofragmentation progresses and molecular weight decreases, the chemical composition and roughness of the petropolymer surfaces are altered. These modifications can stimulate bacterial EPS production, leading to thicker and more robust biofilms. The resulting biofilm structure may be more stable with increased interactions between bacterial cells and the degraded polymer surface, making the biofilm more resistant to removal by physical or chemical means.

### 3.3 Review of Key Factors Influencing Biodegradation during the Third Step of Biofilm Formation: Disaggregation and Detachment (Figure 2e)

The final stage of biodegradation involves the complete breakdown of plastic fragments into carbon dioxide, water, and biomass (Figure 2e). Fragment size has been shown to significantly influence the rate of the final stage of biodegradation. Studies have reported that polymer particles below approximately 300 μm exhibit enhanced microbial colonization and enzymatic activity compared to larger counterparts.^72^ For example, polyethylene fragments in the 100–300 μm range support higher biofilm density and faster surface oxidation, attributed to their increased surface area-to-volume ratio and improved nutrient diffusion. While precise thresholds may vary depending on the polymer type and environmental conditions, incorporating such size considerations into the material design could improve degradation efficiency in practical settings.

Additionally, while biofilms are essential for initiating plastic degradation, disaggregation and detachment play crucial roles in spreading biodegraded plastic fragments and microbial cells into the environment.^12^ Detached biofilm fragments, containing cells and enzymes, can colonize new surfaces or degrade dispersed plastic fragments, facilitating further biodegradation. Detachment helps prevent overcrowding and resource depletion within the biofilm. This dynamic nature ensures the constant renewal of active biofilm layers, enhancing the overall plastic degradation efficiency. In some environments (e.g., industrial settings or water systems), biofilm detachment can pose challenges as detached biofilm fragments can lead to contamination or clogging of equipment. Controlling biofilm formation and disaggregation is crucial for balancing biodegradation with practical management needs.

## 4 Discussion: Engineering Implication and Challenge

One of the intrinsic challenges lies in the molecular structure of petropolymers themselves.^73^ The high molecular weight, crystalline structure, and hydrophobicity of petropolymers collectively present substantial barriers to microbial colonization and enzymatic degradation.^74^ Unlike other polymers, which have clear enzymatic targets, polyolefins lack easily accessible functional groups, complicating enzymatic recognition and cleavage efficiency.^74^

Another challenge is that biofilms are not uniform entities.^75^ They can house a myriad of microbial species, each contributing differently to the degradation process.^76^ While some strains might exhibit petropolymer degradation potential in the laboratory, their real-world efficiency remains uncertain due to the complexities of biofilms.^76^

Standardizing parameters that are important to biofilm-mediated degradation across studies has also been a challenge, leading to inconsistencies in observed degradation rates.^77^ In nature, both biofilm formation and polymer degradation can be easily affected by factors such as air moisture, pH, temperature, and nutrient availability.^78^ In addition, quantifying degradation is also not straightforward.^77^ Different studies might employ varying metrics, from weight loss to changes in molecular weight or surface morphology. This diversity in methodologies can lead to discrepancies in reported results, fueling debates about the actual potential of biofilm-mediated petropolymer degradation. There is also ongoing scholarly debate regarding the precise role of biofilms in enhancing or inhibiting enzymatic plastic degradation. Some studies suggest that biofilm formation facilitates substrate localization and enzyme retention, improving degradation efficiency, while others argue that biofilms can act as diffusion barriers, reducing access to the polymer surface. Additionally, there is inconsistency in reported degradation rates across studies due to variable environmental conditions and differences in experimental protocols. Contradictions also persist regarding the enzymatic activity of laccases and peroxidases on recalcitrant plastics such as polyethylene under environmentally relevant conditions. These controversies highlight the need for standardized biodegradation assays and deeper mechanistic research to reconcile these differing perspectives.

While enzymatic methods offer a sustainable alternative to chemical processes, the costs associated with enzyme production and the potential for incomplete degradation present challenges that must be addressed to ensure that the technology is both environmentally beneficial and economically feasible. The current status is that crude laccase,^45^ peroxidase,^79^ and esterase^70^ extracts have shown the modification of PE and PS surfaces and degrade the ester bonds in PU. However, no isolated enzymes have been confirmed to fully degrade petropolymers. PETase and MHETase are the most advanced areas, with several recombinant and engineered enzymes capable of depolymerizing PET into terephthalic acid (TPA) and ethylene glycol (EG).^80,81^ Recent advances in the field have demonstrated the potential of both bacterial strains and isolated enzymes in plastic degradation systems. While the presence of live biofilm-forming bacteria offers localized and sustained enzymatic activity, isolated enzymes, such as PETase and MHETase, have been engineered for improved thermostability, substrate specificity, and catalytic turnover, making them viable candidates for direct application in controlled environments. However, each approach presents limitations: biofilm systems may lead to clogging in reactors, uneven surface colonization, and undesired biodeterioration of adjacent infrastructure, while free enzymes often face challenges such as low stability and high cost. These dual strategies—microbial consortia and purified enzyme systems—are currently being optimized to balance efficacy, scalability, and environmental applicability.

Technological constraints in designing bioreactors or environments that can sustain biofilm growth while ensuring optimal petropolymer degradation add layers of complexity to the research.^82^ The successful application of enzymatic biodegradation at an industrial scale involves several engineering challenges, primarily centered around enzyme stability, efficiency, and integration into existing waste management systems. Enzyme stability under industrial conditions is another critical issue as enzymes are sensitive to temperature fluctuations, pH changes, and inhibitors, which can reduce their effectiveness over time. Engineering solutions, such as robust enzyme design or enzyme immobilization techniques, can enhance stability. Additionally, ensuring that the degradation process is efficient enough for economic viability requires optimizing reaction conditions like temperature and enzyme concentration and developing continuous processing systems capable of handling large volumes of waste polymers. Another challenge is the integration of enzymatic degradation into current waste management infrastructure, which may involve retrofitting or developing new facilities to accommodate enzymatic processing, while ensuring that degradation products are managed appropriately to prevent secondary pollution.

## 5 Conclusions and Future Directions

The development of biodegradable petropolymers represents a promising avenue for advancing sustainable waste management and enhancing plastic circularity. By incorporating biodegradable components into conventional plastics, researchers aim to improve microbial susceptibility, making synthetic polymers more amenable to enzymatic breakdown postdisposal.^83^ However, optimizing this approach requires a deeper understanding of enzyme–plastic interactions, the role of biofilms, and the environmental factors that govern the degradation efficiency.

Enzymes, acting as catalysts in polymer breakdown, facilitate hydrolysis and oxidation reactions, converting complex polymers to monomers and oligomers. Enzyme kinetics are characterized by the Michaelis–Menten equation:

1 $$\frac{1}{-r_{s}}=\frac{1}{V_{max}}+\frac{K_{M}}{V_{max}}\;*\;\frac{1}{\left[S\right]}$$

where -*r*_s_ is the rate of disappearance of substrate (S), *V*_max_ is the maximum rate of reaction for a given total enzyme concentration, *K*_M_ is the Michaelis constant or affinity constant, and [*S*] is the concentration of substrate (S) in the enzyme solution with a known enzyme concentration.^84^ In Table S3, we summarize the kinetics of enzymes listed in Table S2 in degrading small molecules that have been characterized previously. Table S3 suggests that the mechanisms of enzyme action, which include hydrolysis and oxidation, depend on factors such as the type of polymer, specific enzymes, and environmental conditions such as pH and temperature.

While the Michaelis–Menten equation provides insight into enzyme kinetics and extensive research has been conducted on the role of enzymes in the biodegradation of synthetic polymers, facilitating the breakdown of complex polymer chains into simpler molecules (Table S2), their kinetics in degrading long-chain polymers such as petropolymers remain unknown, which deserves further investigation. For example, polymers with ester linkages, such as polyethylene terephthalate (PET), are particularly susceptible to enzymatic hydrolysis, resulting in the formation of monomers, oligomers, and other low-molecular-weight byproducts.^85^ The efficiency of this process is influenced by the affinity between the enzyme and polymer substrate as well as the maintenance of optimal environmental conditions. The biodegradation products, which vary depending on the polymer and enzyme involved, include monomers like terephthalic acid and ethylene glycol, oligomers that may require further degradation, and other byproducts that can either be benign or need additional processing.^86^ These products have significant implications for environmental impact, as they may either accumulate and pose risks or serve as substrates for further microbial degradation, ultimately leading to complete mineralization into CO_2_ and water. However, the kinetics of these enzymes in degrading petropolymers remain unknown.

The efficiency of enzymatic biodegradation is also highly dependent on polymer composition, with PET undergoing hydrolysis into terephthalic acid and ethylene glycol, whereas polyolefin degradation often results in partially oxidized hydrocarbons, requiring further bioprocessing to achieve complete mineralization into CO_2_ and water. Future research should focus on improving biodegradation efficiency through computational models that integrate biofilm growth dynamics and enzyme kinetics as well as the implementation of real-time spectroscopic techniques such as FTIR and Raman spectroscopy to track polymer breakdown at the molecular level. Additionally, standardizing degradation metrics across different environmental conditions will enable better cross-study comparisons, ensuring reproducibility and accelerating industrial applications. By integrating biofilm-mediated enzymatic degradation with existing recycling technologies, researchers can develop multistage waste treatment strategies that offer a scalable and sustainable solution to global plastic pollution.

A wide array of oxidative and hydrolytic enzymes, as summarized in Table 1, Figure 2, and Table S2, play essential roles in biofilm-mediated biodegradation. Each enzyme exhibits specificity toward particular chemical bonds or functional groups. Understanding the interactions between these enzymes and petropolymers can guide the rational design of enzyme-responsive smart materials in several key directions: (1) Enzyme-triggered self-assembly and disassembly: Smart polymers can be engineered to self-assemble into nanostructures that disassemble upon exposure to target enzymes. This strategy has been successfully employed in controlled drug delivery systems, where enzymatic activity induces structural disintegration and payload release.^87^ (2) Incorporation of enzyme-cleavable linkers: By embedding specific peptide sequences or chemical bonds that serve as substrates for target enzymes, polymers can be tailored for precise degradation and controlled release. These cleavable motifs can be designed to respond to a broader range of enzymes beyond those listed in Tables 1 andS2, expanding the functional versatility of the material. In conclusion, leveraging enzyme-specific interactions in material design holds immense potential for developing responsive, tunable systems with applications in environmental remediation, targeted delivery, and smart biomaterials.

## Supplementary Material



## Notes

Table S1. List of biofilm enhanced biodegradation.Table S2. Summary of the enzymes for the biodegradation of petropolymers and the prediction of their effects on biofilm formation during biodegradation.Table S3. Summary of enzyme kinetics in degrading small molecules (PDF)
