# Supplementary material for: Harnessing Biofilm-Mediated Plastic Biodegradation: Innovating Smart Material Design
Source: ACS Appl Eng Mater. 2025 Jul 2;3(7):1915–26. doi: 10.1021/acsaenm.5c00179 (PMC12305493; doi:10.1021/acsaenm.5c00179)
Supplement: Supplementary file 1 [file em5c00179_si_001.pdf]

Supporting Information

Harnessing Biofilm-Mediated Plastic Biodegradation: Innovating Smart Material Design

Kaitlyn Benes[1], Madison Liguori[1], Cody J. Velikaneye[1], Sarah Kispert[1], Alexis Pishnyuk[1], Eddie Luzik[1], Hao Sun[1], Dequan Xiao[1], Huan Gu[1,*]

[1]Department of Chemistry & Chemical Engineering and Biomedical Engineering, Tagliatela College of Engineering, University of New Haven, West Haven, CT 06516, United States

*Corresponding author

Huan Gu: Phone: 203-932-7000. Email: hgu@newhaven.edu

Table S1. List of biofilm enhanced biodegradation.

	Petropolymers	Microorganisms	Biofilm	Level of biodegradation	Techniques used	Mechanism/Products of biodegradation	Ref.
Film	• LDPE<br>• HDPE	• Bacillus safensis	• The hydrophobicity degree of the cell surface helps microorganisms easily adhere to the surface of nonpolar materials such as polyethylene.<br>• Non-polar polymers such as LDPE are hard to decompose because of their hydrophobic surfaces.<br>• Plasma-activated bacteria can increase their attachment onto non-polar polymers.	• Plasma-activated bacteria could induce cracks, holes, and roughness on the surface of LDPE films over 90 days.<br>• Over 30 days; the LDPE film lost 13.40 ± 0.013% and 27.78 ± 0.014% of its mass by BS-10L and plasma-treated BS-10L, respectively.	• Cultivation method<br>• 16S rRNA sequencing<br>• SEM, FTIR, AFM, and XPS<br>• Plasma-activated bacteria<br>• BATH assay for measuring cell surface hydrophobicity	• New peaks of the C=O and C-O groups in the plasma-treated LDPE film.<br>• Plasma-activated BS-10L could accelerate decomposition by oxidation by increasing the carbonyl group of the PE film.	[1]<br>Ji, et al. 2024
		• Bacillus mycoides and Bacillus subtilis		• LDPE & HDPE were exposed outdoor to the Niger Delta mangrove soil for 24 hours.<br>• Biodegradation in Erlenmeyer flasks by the bacteria after 60 days of incubation ranged between 8.41%-23.15%.	• FTIR	• Introduction of carbonyl groups after natural weathering, which decreased after microbial treatment. Decrease in carbonyl index ranged between 10.5%-13.7%.<br>• The carbonyl band corresponds to the ketone and ester carbonyl groups and it is a typical product of oxidative degradation of PE (Gilan et al. 2004; Hadad et al., 2005).<br>• These compounds which are degradation products along with the oxidized low molecular weight polymer have been reported to be assimilated by microorganisms (Hakkarainen & Albertsson, 2004).	[2]<br>Ibiene et al., 2013
		• Bacillus sphericus GC subgroup IV<br>• Bacillus cereus subgroup A	• Decrease in contact angle indicated that the surfaces turned more hydrophilic after exposure.	• The weight loss of the thermally treated LDPE and HDPE samples were about 19% and 9% respectively, and unpretreated samples were 10% and 3.5% respectively with B. sphericus in 1 year. Weight loss of unpretreated starch-blended LDPE was 25% with B. cereus. Tensile strength of thermally pretreated LDPE and HDPE and unpretreated	• FTIR<br>• AFM	• FTIR spectrum showed that initially carbonyl index increased, probably due to oxidation by dissolved oxygen (abiotic factor).<br>•	[3]<br>Sudhakar et al, 2008

				starchblended LDPE decreased by 27%, 14.8% and 30.5%, respectively, with *B. sphericus* and the corresponding decrease in crystallinity was 8%, 2.2% and 8.5%, respectively.			
	• LDPE	• Micrococcus luteus IRN20<br>• Cupriavidus necator H16		• The percent decrease in LDPE mass ranged from 18.9% ± 0.72% for M. luteus IRN20 to 33.7% ± 1.2% for *C. necator* H16 after 21 days incubations.		• Linear alkane hydrolysis products from LDPE degradation were detected in the culture media, and the carbon chain lengths of the hydrolysis products detected varied, depending on the species of bacteria.<br>• We also determined that C. necator H16 produced short-chain-length polyhydroxyalkanoate biopolymers, while Pseudomonas putida LS46 and Acinetobacter pittii IRN19 produced mediumchain-length biopolymers while growing on polyethylene powder.<br>• Cupriavidus necator H16 accumulated poly (3-hydroxybutyrate-co-3-hydroxyvalerate) (PHB-V) polymers to 3.18% ± 0.4% of cdm.<br>• The monomer composition of the PHB-V was 94.9% ± 0.61% 3-hydroxybutyrate and 5.03% ± 0.56% 3-hydroxyvalerate.	[4]<br>Montazer, et al. 2019
	• PP	• B. flexus + P. azotoformans (B1)<br>• B. flexus + B. subtilis (B2)	• polymers become hydrophilic	• Thermo gravimetric analysis also revealed that UV treated PP exposed to B. flexus + P. azotoformans combination for one year exhibited maximum degradation (22.7%).<br>• The gravimetric weight loss method showed 1.95% weight loss followed by 1.45% with B. flexus + B. subtilis.	• FTIR<br>• TGA<br>• CLSM<br>• SEM	• Treatment strategy might play a major role in enhanced degradation of PP	[5]<br>Aravintha, et al.

	• PE	• Rhodococcus ruber C208		• Biofilm-producing strain (C208) of Rhodococcus ruber degrades PE at a rate of 0.86% per week ($r^2 = 0.98$).	• SEM<br>• Fluorescent microscopy	• Strain C208 adheres to PE immediately upon exposure to the polyolefin.<br>• The biofilm allowed a high viability even after 60 days of incubation.	[6] Sivan et al, 2006
		• Rhodococcus ruber C208	• A strain of Rhodococcus ruber (C208) displayed high hydrophobicity and formed a dense biofilm on the surface of polyethylene films while utilizing the polyolefin as carbon and energy sources.		• Crystal violet<br>• SEM	• Proteinase K enhanced biofilm formation, which was robust and multilayered.	[7] Gilan et al, 2013
		• Bacterial and fungal communities at the Belgian part of the 27 North Sea<br>• *Cladosporium cladosporioides*, *Fusarium* 439 *redolens*, and at lower abundance *Mortierella alpine*	• none of the plastics exposed to offshore conditions displayed the typical 32 signature of a late stage biofilm		• ITS2 30 metabarcoding<br>• 16S rDNA sequencing<br>• Principal Coordinate Analysis (PCoA)<br>• PERMANOVA analysis	• Sample type (plastic sheet, dolly rope, seawater 282 or sediment; PERMANOVA, p<0.001); environment (harbor or offshore) (PERMANOVA, p <0.001); and time of exposure (PERMANOVA, p<0.001), significantly affect 283 the bacterial community 284 composition, with highly significant interaction effects (all interaction effects p<0.001) between all three 285 factors.	[8] Tender, et al.

	• PP<br>• pro-oxidant blended (MI-PP)<br>• starch blended polypropylenes (ST-PP)	• Phanerochaete chrysosporium NCIM 1170<br>• Engyodontium album MTP091 (F2).	• UV treatment oxidizes the surface more effectively than the thermal treatment.<br>• The surfaces of treated PP films shows cracks and grooves due to the abiotic treatment.	• About 18.8 and 9.42 % gravimetric weight loss and 79 and 57% TGA weight loss (at 400oC) were observed with UV pretreated MI-PP in one year with F2 and F1 strains respectively.	• SEM<br>• Thermal Analyser (TA)<br>• GC–MS equipped with HP<br>• FTIR	• The amount of lacasse produced by the organism and biomass attached on the polymer surface are corelated with TGA weight loss(0.6-0.93). The formation of extractable oxygenated compounds and unoxidized low-molecular weight hydrocarbons are high in pretreated and blended samples.<br>• Pretreatment and blending strategy proves to accelerate the biodegradation process. Metal ions are a more effective degradant than starch under UV conditions.	[9]<br>Jeyakumar, et al.
	• PS	• Rhodococcus ruber C208	• Most of the bacterial cells adhered to the polystyrene surface within few hours, forming a biofilm.	• Extended incubation of the biofilm for up to 8 weeks resulted in a small reduction in the polystyrene weight (0.8% of gravimetric weight loss).	• Crystal violet (CV)<br>• Fluorescent redox probe 5-cyano-2,3-ditolyl tetrazolium chloride (CTC).<br>• SEM	• Addition of mineral oil (0.005% w/v), but not non-ionic surfactants, increased the biofilm.<br>• The respiration rate, of the biofilm, exhibited a pattern similar to that of the biofilm growth.	[10]<br>Mor et al, 2008
Microplastics	• LDPE<br>• PE with pro-oxidant (OXO)<br>• Artificially aged OXO (AA-OXO)<br>• poly(3-hydroxybutyrate-co-3-hydroxyvalerate) (PHBV)	• Gammaproteobacteria (Alcanivorax sp., Aestuariicella hydrocarbonica, Alteromonas sp., and Thalassolituus sp. Marinobacter sp. and Maricurvus sp.)	• PE, OXO, and AA-OXO showed a smooth surface and no surface modification during biodegradation.<br>• PHBV presented a spherulitic structure of about 20 µm on their surface and their roughness went through swelling and erosion during 45 days of incubation in seawater (SW).	• Seawater, Mediterranean Sea, France	• AFM<br>• Contact angle<br>• Fluorescent microscopy<br>• Flow cytometry<br>• Bacterial production<br>• 16S rRNA Sequencing<br>• RMS (roughness, nm) and carbonyl index (biodegradation, CI)	• Although the microbial composition on PE, OXO, AA-OXO, and PHBV are similar, the distribution of species varies significantly.	[11]<br>Dussud, et al. 2018
	• PE<br>• PP	• Acetobacteraceae (Roseococcus sp.)<br>• Rhodanobacteraceae (Luteolibacter sp.)		• Freshwater	• 16S rRNA Sequencing<br>• Functional profiles (KEGG) predicted by Tax4Fun	• Significantly higher abundances of Pirellulaceae, Phycisphaerales, Cyclobacteriaceae, and Roseococcus were observed on the microplastic substrates compared with the natural substrates.<br>• The pathways of amino acid metabolism and metabolism of cofactors and vitamins were increased in biofilms on the microplastic substrates.	[12]<br>Miao et al., 2019

		• Rhodothermaceae (Rubrimonas sp., Thalassobius sp., Albidovulum sp., Rhodovulum sp., & Roseovarius sp.)<br>• Hyphomonadaceae (Hyphomonas sp.)<br>• Alteromonadaceae (Alteromonas sp.)			• SEM<br>• Amplicon Pyrotag Sequencing.<br>• Rama microscopy<br>•	• Pits visualized in the PMD surface conformed to bacterial shapes suggesting active hydrolysis of the hydrocarbon polymer.<br>• Small-subunit rRNA gene surveys identified several hydrocarbon-degrading bacteria, supporting the possibility that microbes play a role in degrading PMD.	[13] Zettler et al, 2013
	• Polyvinyl alcohol (PVA)	• Alteromonadaceae (Alteromonas sp.)<br>• Alphaproteobacteria (Rhodobacter sp.)	• A comparison of the colonization patterns on the test surfaces indicated that the early bacterial community succession rate and/or direction may be influenced by surface physicochemical properties.		• Amplified rRNA Gene Restriction Analysis<br>• Phylogenetic analyses of 16s rRNA genes	• 22 of the 26 clones sequenced were affiliated with the Roseobacter subgroup and Two clones were affiliated with the Alteromonas group.<br>• Roseobacter subgroup are ubiquitous and rapid colonizers of surfaces in coastal environments.	[14] Dang & Lovell et al. 2000
	• Poly(ethylene terephthalate) (PET)	• Flavobacteriaceae (Tenacibaculum sp.)<br>• Cryomorphaceae (Crocinitomix sp.)		• Up to 5-6 weeks at North Sea.	• SEM<br>• DNA extraction and 16s and 18s rRNA sequencing<br>• Beta Diversity Analysis<br>• Significantly discriminant OTUs PET community correlation analyses	• PET-colonizing taxa are all known to degrade complex carbon substrates and diatoms.<br>• PET-colonizing microbial communities differed significantly from free-living communities, but from particle associated (>3 µm) or those inhabiting glass substrates.	[15] Oberbeckmann et al, 2016
	• PE<br>• PS<br>• PP	• Sphingomonadaceae (Parasphingopyxis sp.)<br>• Rhodothermaceae (Roseovarius sp.)		• Slovenian coast of the North Adriatic sea	• ATR-FTIR<br>• DNA isolation<br>• Phylogenetic analysis	• 28 bacterial species were identified on the microplastics particles including Aeromonasspp. and hydrocarbon-degrading bacterial species	[16] Virsek et al, 2017

		• Pseudorueger ia sp.<br>• Erythrobacter aceae (Erythrobacte r sp.)					
		• Bacillariophy ceae (Diatoms Cocconeis sp., Amphora sp., Cymbella sp., Achananthes sp, Haslea sp, Mastogloia sp., Grammatoph ora sp., Thalassione ma sp., Nitzschia sp.)<br>• Dinoflagellat a (Ceratium sp.)	• A variety of plastic surface microtextures, including pits and grooves conforming to the shape of microorganisms was identified, suggesting that biota may play an important role in plastic degradation.	• Coastal regions of Australia	• SEM<br>FTIR		[17]<br>Reisser et al, 2014
		• Epsilonprote obacteria (Arcobacter sp.)<br>• Gammaprote obacteria (Aeromonas sp.)		Chicago river, Chicago, USA	• SEM<br>• high-throughput sequencing<br>•	• Pseudomonas occupies 17.81% of the taxon on microplastic surface.	[18]<br>McCormick et al, 2014
		• Erythrobacter aceae<br>• Sphingomon adaceae<br>• Comamonad aceae<br>• Blastocatella ceae<br>• Rhodobacter acea		• Yangtze Estuary, China	• Spectroscopic analysis<br>• Micro-FTIR<br>• DNA extraction and sequencing<br>• MOTHUR v.1.33.3 software KEGG Orthology	• Microbial communities colonized the surface of microplastic particles in the intertidal environments around Yangtze estuary clustered by geographical locations rather than by polymer types.<br>• Specific metabolic pathways were enriched within the plastic-colonizing bacterial communities.	[19]<br>Jiang et al, 2018

		• Erythrobacteraceae<br>• Moraxellaceae<br>• Planococcaceae					
		• ambient bacterioplankton assemblage from the Baltic Sea		• Baltic Sea	• Physicochemical properties (compression, crystallinity, surface chemistry, hydrophobicity, and surface topography)<br>• 16S rRNA gene sequencing	• Significant changes in PE crystallinity, PP stiffness, and PS maximum compression were observed as a result of exposure to bacteria.<br>• PE samples having significantly higher contribution of Sphingobium, Novosphingobium, and uncultured Planctomycetaceae compared to the other test materials, whereas PP and PS samples had significantly higher abundance of Sphingobacteriales and Alphaproteobacteria.	[20] McGiveny et al.
	• PE	• Comamonas<br>• Delftia,<br>• Stenotrophomonas	• Our results indicate that these microbes are capable of degrading unpretreated PE of very high molecular weight (191,000 g.mol-1) and survive for long periods under this condition.<br>• These bacterial strains from the genera Comamonas, Delftia, and Stenotrophomonas showed metabolic activity and cellular viability after a 90-day incubation with PE as the sole carbon source.<br>• Considerable nanoroughness shifts and vast damages to the micrometric surface were confirmed by AFM and SEM.	• Cerrado soil	• ATR<br>• FTIR<br>• AFM<br>• SEM<br>• Rama microscopy	• Nitrogen metabolism is involved in the chemical modification of PE.<br>• ATR/FTIR indicated that biodegraded PE undergone oxidation, vinylene formation, chain breakage, among other chemical changes.<br>• Phase imaging revealed a 46.7% decrease in the viscous area of biodegraded PE whereas Raman spectroscopy confirmed a loss in its crystalline content, suggesting the assimilation of smaller fragments.	[21] Peixoto et al., 2016

Table S2. Summary of the enzymes for the biodegradation of petropolymers and the prediction of their effects on biofilm formation during biodegradation.

Enzymes	Effects on biofilm formation:<br>(+): enhancing/promoting<br>(-): inhibiting/decreasing<br>(n): either (+) or (-) depending on the circumstances	Microorganisms	Petro-polymers	Ref
Cutinase	(+): increasing surface roughness by creating pits, cracks, and other surface irregularities as polymer is broken down (Fig. 2c-e).<br>(+): increasing surface hydrophobicity.<br>(+): enhancing EPS production by providing a steady supply of nutrients.	*Thermobifida fusca WSH03-11*	PE	*22, 29*
		Cutinase *(LCC^{WCCG}): Leaf-branch compost metagenome*	PET	*30*
		Cutinase (PE-H^{Y250S}): *Pseudomonas aestusnigri* VGXO14T		*31*
		Cutinase (TfCut^{WA}): *Thermobifida fusca KW3*	PU	*32*
Lipase	(+): carbon metabolism.	*Halomonas sp*	PE	*22, 33*
		Pseudomonas spp.<br>*Bacillus spp.*	PS	*34*
Esterase	(+): formation of surface cracks and deep holes (Fig. 2c-e).	*Exiguobacterium sp.*<br>*Halomonas sp.*<br>*Ochrobactrum sp.*	PE	*22, 35*
		Pseudomonas spp.<br>*Bacillus spp.*	PS	*34*
		Comamonas acidovorans TB-35	PU	*36*
BHETase	(+): increasing surface hydrophobicity.<br>(+): enhancing EPS production by providing a steady supply of nutrients.	BHETase (DBsEst): *Bacillus subtilis PET-86*	PET	*37*
		BHETase (ΔChryBHETase): *Chryseobacterium sp. PET-29*		
MHETase (MHETase$^{R411K/S416A/F424I}$)	(+): increasing surface hydrophobicity.<br>(+): enhancing EPS production by providing a steady supply of nutrients.	*Ideonella sakaiensis 201-F6*		*44*

Table S3. Summary of enzyme kinetics in degrading small molecules.

	Substrate	Condition	Km	Vmax	Ref
Laccase	2,2'-azino-bis(3-ethylbenzothiazoline-6-sulfonic acid) (ABTS)	65 °C, pH 4.0	1.42 mM	184.84 U·mg^{-1}	45
		pH 5.0	0.38 mM; 3.97 mM; 0.46 mM	71.42 U· ml^{-1}; 148.8 U· ml^{-1}; 23.42 U· ml^{-1}	46
Cutinase	4-nitrophenyl (16-methyl sulfone ester) hexadecanoate (pNMSEH)		1.8 mM	10.5 μmol ·min^{-1}· l^{-1}	47
Lipase	p-nitrophenyl palmitate	pH 7.0, 45 °C	7.37 mM	25.91 μmol·min^{1}·mg^{1}	48
		pH 8.0, 45 °C	0.33	188 μmol·min^{1}·mg^{1}	49
Esterase	1-naphthyl acetate	37 °C	28 μM	6.0 μmol·min^{1}·mg^{1}	50
	p-nitrophenyl butyrate	pH 8.0, 37 °C	11 μM	131.6 μmol·min^{1}·mg^{1}	51
Maganese peroxidase	phenol red	35 °C, pH of 4.5	8 μM	111.14 μmol·min^{1}·mg^{1}	52
	$MnSO_4$	pH 5, 50 °C	70 μM	540 μmol·min^{1}·mL^{1}	53
Soybean peroxidase	Veratryl alcohol	pH < 4.0,	78 μM	0.58 μmol·min^{1}·mL^{-1}	54
	p-Cresidine	pH 7.4, 23 °C	79.1 ± 1.0 μM	0.0690 ± 0.0060 μM·s^{-1}	55
	4,4′-oxydianiline		41.5 ± 5.8 μM	0.0647 ± 0.0100 μM·s^{-1}	
	4-chloro-o-toluidine		691 ± 53 μM	0.293 ± 0.070 μM·s^{-1}	
	4,4′-methylenebis (2-chlororaniline)		1.70 ± 0.14 μM	0.0145 ± 0.0004 μM·s^{-1}	
	4,4′-methylenedianiline		979 ± 80 μM	0.536 ± 0.030 μM·s^{-1}	56
	4,4′-thiodianiline		15.0 ± 3.0 μM	0.0234 ± 0.0020 μM·s^{-1}	
	Acid Blue		21.0 ± 2.8 μM	0.110 ± 0.006 μM·s^{-1}	57
	Direct Black 38		36.0 ± 4.3	0.190 ± 0.009	

			μM	$\mu M \cdot s^{-1}$	
	Crocein Orange G		4.70 ± 0.45 μM	0.0140 ± 0.0005 $\mu M \cdot s^{-1}$	58
Dioxygenase (HIS1)	Linoleic acid	pH 10	0.48 mM	1.74 $\mu mol \cdot min^{-1} \cdot mg^{-1}$	59
	Catechol	pH 8.0, 40 °C	12.8 μM	1,218.8 $U \cdot mg^{-1}$	60
Hydroquinone peroxidase	Hydroquinone	pH 6, 35 °C	0.045 nM	0.5 $\mu mol \cdot min^{-1} \cdot mg^{-1}$	61
PETase	pNP-C6	pH 4–10, 4–65 °C	0.73 mM	0.86 $nmol \cdot min^{-1} \cdot mg^{-1}$	62
Chitinase	Colloidal chitin	pH 6.7	2.92 mM	4.26 $\mu mol \cdot min^{-1} \cdot mg^{-1}$	63
	Chitin	pH 3–9, 25 °C	8.3 mg/ml	2.4 $mmol \cdot min^{-1}$	64

References:

(1) Ji, S. H.; Yoo, S.; Park, S.; Lee, M. J. Biodegradation of Low-Density Polyethylene by Plasma-Activated *Bacillus* Strain. *Chemosphere* **2024**, *349*, 140763. DOI: 10.1016/j.chemosphere.2023.140763.

(2) Ibiene, A. A.; Stanley, H. O.; Immanuel, O. M. Biodegradation of Polyethylene by *Bacillus sp.* Indigenous to the Niger Delta Mangrove Swamp. *Nig. J. Biotech.* **2013**, *26*, 68-79.

(3) Sudhakar, M.; Doble, M.; Murthy, P. S.; Venkatesan, R. Marine Microbe-Mediated Biodegradation of Low- and High-Density Polyethylenes. *Int.l Biodeterior. Biodegrad.* **2008**, *61*, 203–213.

(4) Montazer, Z.; Najafi, M. B. H.; and Levin, D.B.; Microbial Degradation of Low-Density Polyethylene and Synthesis of Polyhydroxyalkanoate Polymers. *Can. J. Microbiol.* **2019**, *65*, 224–234.

(5) Aravinthan, A.; Arkatkar, A.; Juwarkar, A. A.; Doble, M. Synergistic Growth of *Bacillus* and *Pseudomonas* and its Degradation Potential on Pretreated Polypropylene. *Prep. Biochem. Biotechnol.* **2016**, *46* (2), 109-115. DOI: 10.1080/10826068.2014.985836.

(6) Sivan, A.; Szanto, M.; Pavlov, V. Biofilm Development of the Polyethylene-Degrading Bacterium *Rhodococcus ruber*. *Appl. Microbiol. Biotechnol.* **2006**, *72*, 346–352.

(7) Gilan, I.; Sivan, A. Effect of Proteases on Biofilm Formation of the Plastic-Degrading Actinomycete *Rhodococcus ruber* C208. *FEMS Microbiol. Lett.* **2013**, *342* (1), 18-23. DOI: 10.1111/1574-6968.12114.

(8) Tender, C.D.; Devriese, L.I.; Haegeman, A.; Maes, S.; Vangeyte, J.; Cattrijsse, A.; Dawyndt, P.; Ruttink, T. The Temporal Dynamics of Bacterial and Fungal Colonization 1 on Plastic Debris in the North Sea. *Env. Sci. Technol.* **2017**, *51* (13), 7350-7360.

(9) D. Jeyakumar; J. Chirsteen; Mukesh, D. Synergistic Effects of Pretreatment and Blending on Fungi Mediated Biodegradation of Polypropylenes. *Bioresour. Technol.* **2013**, *148*, 78-85.

(10) Mor, R.; Sivan, A. Biofilm Formation and Partial Biodegradation of Polystyrene by the Actinomycete *Rhodococcus ruber*. *Biodegradation* **2008**, *19*, 851–858.

(11) Dussud, C.; Hudec, C.; George, M.; Fabre, P.; Higgs, P.; Bruzaud, S.; Delort, A. M.; Eyheraguibel, B.; Meistertzheim, A. L.; Jacquin, J.; et al. Colonization of Non-biodegradable and Biodegradable Plastics by Marine Microorganisms. *Front. Microbiol.* **2018**, *9*, 1571. DOI: 10.3389/fmicb.2018.01571.

(12) Miao, L.; Wang, P.; Hou, J.; Yao, Y.; Liu, Z.; Liu, S.; Li, T. Distinct Community Structure and Microbial Functions of Biofilms Colonizing Microplastics. *Sci. Total Environ.* **2019**, *650* (Pt 2), 2395-2402. DOI: 10.1016/j.scitotenv.2018.09.378.

(13) Zettler, E. R.; Mincer, T. J.; Amaral-Zettler, L. A. Life in the “Plastisphere”: Microbial Communities on Plastic Marine Debris. *Env. Sci.Technol.* **2013**, *47* (13), 7137–7146

(14) Dang, H.; Lovell, C. R. Bacterial Primary Colonization and Early Succession on Surfaces in Marine Waters as Determined by Amplified rRNA Gene Restriction Analysis and Sequence Analysis of 16S rRNA genes. *Appl. Environ. Microbiol.* **2000**, *66* (2), 467-475. DOI: 10.1128/AEM.66.2.467-475.2000.

(15) Oberbeckmann, S.; Osborn, A. M.; Duhaime, M. B. Microbes on a Bottle: Substrate, Season and Geography Influence Community Composition of Microbes Colonizing Marine Plastic Debris. *Plos One* **2016**, *11* (8), e0159289.

(16) Viršek, M. K.; Lovšin, M. N.; Koren, Š.; Kržan, A.; Peterlin, M. Microplastics as a Vector for the Transport of the Bacterial Fish Pathogen Species *Aeromonas salmonicida*. *Mar. Pollut. Bull.* **2017**, *125* (1-2), 301-309. DOI: 10.1016/j.marpolbul.2017.08.024.

(17) Reisser, J.; Shaw, J.; Hallegraeff, G.; Proietti, M.; Barnes, D. K.; Thums, M.; Wilcox, C.; Hardesty, B. D.; Pattiaratchi, C. Millimeter-sized marine plastics: a new pelagic habitat for microorganisms and invertebrates. *PLoS One* **2014**, *9* (6), e100289. DOI: 10.1371/journal.pone.0100289.
(18) McCormick, A.; Hoellein, T. J.; Mason, S. A.; Schluep, J.; Kelly, J. J. Microplastic is an Abundant and Distinct Microbial Habitat in an Urban River. *Environ. Sci. Technol.* **2014**, *48* (20), 11863-11871. DOI: 10.1021/es503610r.
(19) Jiang, P.; Zhao, S.; Zhu, L.; Li, D. Microplastic-Associated Bacterial Assemblages in the Intertidal Zone of the Yangtze Estuary. *Sci. Total. Environ.* **2018**, *624*, 48-54. DOI: 10.1016/j.scitotenv.2017.12.105.
(20) McGivney, E.; Cederholm, L.; Barth, A.; Hakkarainen, M.; Hamacher-Barth, E.; Ogonowski, M.; Gorokhova, E. Rapid Physicochemical Changes in Microplastic Induced by Biofilm Formation. *Front. Bioeng. Biotechnol.* **2020**, *8*, 205. DOI: 10.3389/fbioe.2020.00205.
(21) Peixoto, J.; Silva, L. P.; Kruger, R. H. Brazilian Cerrado Soil Reveals an Untapped Microbial Potential for Unpretreated Polyethylene Biodegradation *J. Haz. Mat.* **2016**, *11*.
(22) Lee, G. H.; Kim, D. W.; Jin, Y. H.; Kim, S. M.; Lim, E. S.; Cha, M. J.; Ko, J. K.; Gong, G.; Lee, S. M.; Um, Y.; et al. Biotechnological Plastic Degradation and Valorization Using Systems Metabolic Engineering. *Int. J. Mol. Sci.* **2023**, *24* (20). DOI: 10.3390/ijms242015181.
(23) Sivan, A.; Szanto, M.; Pavlov, V. Biofilm Development of the Polyethylene-Degrading Bacterium *Rhodococcus ruber. Appl. Microbiol. Biotechnol.* **2006**, *72* (2), 346-352. DOI: 10.1007/s00253-005-0259-4.
(24) Yao, C.; Xia, W.; Dou, M.; Du, Y.; Wu, J. Oxidative Degradation of UV-irradiated Polyethylene by Laccase-mediator System. *J. Hazard. Mater.* **2022**, *440*, 129709. DOI: 10.1016/j.jhazmat.2022.129709.
(25) Miriam Santo, R. W., Alex Sivan. The Role of the Copper-Binding Enzyme – Laccase – in the Biodegradation of Polyethylene by the Actinomycete R*hodococcus ruber*. *Int. Biodeterior. Biodegrad.* **2013**, *84*, 204-210.
(26) Magnin, A.; Entzmann, L.; Pollet, E.; Avérous, L. Breakthrough in Polyurethane Bio-Recycling: An Efficient Laccase-Mediated System for the Degradation of Different Types of Polyurethanes. *Waste Manag.* **2021**, *132*, 23-30. DOI: 10.1016/j.wasman.2021.07.011.
(27) Yoon, M. G.; Jeon, H. J.; Kim, M. N. Biodegradation of Polyethylene by a Soil Bacterium and AlkB Cloned Recombinant Cell. *J. Bioremediation Biodegrad.* **2012**, *3*, 145.
(28) Zhao, J. C.; Guo, Z.; Ma, X. Y.; Liang, G. Z.; Wang, J. L. Novel Surface Modification of High-Density Polyethylene Films by Using Enzymatic Catalysis. *J. Appl. Polym. Sci.* **2004**, *91*, 3673–3678.
(29) Kong, D.; Wang, L.; Chen, X.; Xia, W.; Su, L.; Zuo, F.; Yan, Z.; Chen, S.; Wu, J. Chemical-Biological Degradation of Polyethylene Combining Baeyer–Villiger Oxidation and Hydrolysis Reaction of Cutinase. *Green Chem.* **2022**, *24*, 2203–2211.
(30) Tournier, V.; Topham, C. M.; Gilles, A.; David, B.; Folgoas, C.; Moya-Leclair, E.; Kamionka, E.; Desrousseaux, M. L.; Texier, H.; Gavalda, S.; et al. An Engineered PET Depolymerase to Break Down and Recycle Plastic Bottles. *Nature* **2020**, *580* (7802), 216-219. DOI: 10.1038/s41586-020-2149-4.
(31) Bollinger, A.; Thies, S.; Knieps-Grünhagen, E.; Gertzen, C.; Kobus, S.; Höppner, A.; Ferrer, M.; Gohlke, H.; Smits, S. H.; Jaeger, K. E. A Novel Polyester Hydrolase from the Marine Bacterium *Pseudomonas aestusnigri*–Structural and Functional Insights. *Front. Microbiol.* **2020**, *11*, 114.

(32) Schmidt, J.; Wei, R.; Oeser, T.; Dedavid E Silva, L. A.; Breite, D.; Schulze, A.; Zimmermann, W. Degradation of Polyester Polyurethane by Bacterial Polyester Hydrolases. *Polymers (Basel)* **2017**, *9* (2). DOI: 10.3390/polym9020065.
(33) Khandare, S. D.; Chaudhary, D. R.; Jha, B. Marine Bacterial Biodegradation of Low-Density Polyethylene (LDPE) Plastic. *Biodegradation* **2021**, *32* (2), 127-143. DOI: 10.1007/s10532-021-09927-0.
(34) Mohan, A. J.; Sekhar, V. C.; Bhaskar, T.; Nampoothiri, K. M. Microbial Assisted High Impact Polystyrene (HIPS) Degradation. *Bioresour. Technol* **2016**, *213*, 204-207. DOI: 10.1016/j.biortech.2016.03.021.
(35) Gao, R.; Sun, C. A Marine Bacterial Community Capable of Degrading Poly(ethylene terephthalate) and Polyethylene. *J. Hazard. Mater.* **2021**, *416*, 125928. DOI: 10.1016/j.jhazmat.2021.125928.
(36) Akutsu, Y.; Nakajima-Kambe, T.; Nomura, N.; Nakahara, T. Purification and Properties of a Polyester Polyurethane-Degrading Enzyme from Comamonas acidovorans TB-35. *Appl. Environ. Microbiol.* **1998**, *64* (1), 62-67. DOI: 10.1128/AEM.64.1.62-67.1998.
(37) Li, A.; Sheng, Y.; Cui, H.; Wang, M.; Wu, L.; Song, Y.; Yang, R.; Li, X.; Huang, H. Discovery and Mechanism-Guided Engineering of BHET Hydrolases for Improved PET Recycling and Ppcycling. *Nat. Commun.* **2023**, *14* (1), 4169. DOI: 10.1038/s41467-023-39929-w.
(38) Iiyoshi, Y.; Tsutsumi, Y.; Nishida, T. Polyethylene Degradation by Lignin-Degrading Fungi and Manganese Peroxidase. *J. Wood Sci.* **1998**, *44*, 222–229.
(39) Yun, S. D.; Lee, C. O.; Kim, H. W.; An, S. J.; Kim, S.; Seo, M. J.; Park, C.; Yun, C. H.; Chi, W. S.; Yeom, S. J. Exploring a New Biocatalyst from *Bacillus thuringiensis* JNU01 for Polyethylene Biodegradation. *Environ. Sci. Technol. Lett.* **2023**, *10*, 485–492.
(40) Tan, Q.; Chen, W.; Liu, H.; Yan, W.; Huang, X.; Li, Y. The Programmed Sequence-Based Oxygenase Screening for Polypropylene Degradation. *J. Hazard. Mater.* **2024**, *465*, 133173. DOI: 10.1016/j.jhazmat.2023.133173.
(41) Nakamiya, K.; Ooi, T.; Kinoshita, S. Non-Heme Hydroquinone Peroxidase from *Azotobacter beijerinckii* HM121. *J. Ferment. Bioeng.* **1997**, *84*, 14–21.
(42) Jolivalt, C.; Madzak, C.; Brault, A.; Caminade, E.; Malosse, C.; Mougin, C. Expression of Laccase IIIb from the White-Rot Fungus *Trametes versicolor* in the Yeast *Yarrowia lipolytica* for Environmental Applications. *Appl. Microbiol. Biotechnol.* **2005**, *66* (4), 450-456. DOI: 10.1007/s00253-004-1717-0.
(43) Son, H. F.; Joo, S.; Seo, H.; Sagong, H. Y.; Lee, S. H.; Hong, H.; Kim, K. J. Structural Bioinformatics-Based Protein Engineering of Thermo-Stable PETase from *Ideonella sakaiensis*. *Enzyme Microb. Technol.* **2020**, *141*, 109656. DOI: 10.1016/j.enzmictec.2020.109656.
(44) Sagong, H. Y.; Seo, H.; Kim, T.; Son, H. F.; Joo, S.; Lee, S. H.; Kim, S.; Woo, J. S.; Hwang, S. Y.; Kim, K. J. Decomposition of the PET Film by MHETase Using Exo-PETase Function. *ACS Catal.* **2020**, *10*, 4805–4812.
(45) Atalla, M. M.; Zeinab, H. K.; Eman, R. H.; Amani, A. Y.; Abeer, A. A. Characterization and Kinetic Properties of the Purified *Trematosphaeria mangrovei* Laccase Enzyme. *Saudi. J. Biol. Sci.* **2013**, *20* (4), 373-381. DOI: 10.1016/j.sjbs.2013.04.001.
(46) Gaur, N.; Narasimhulu, K.; Pydisetty, Y. Biochemical and Kinetic Characterization of Laccase and Manganese Peroxidase from Novel. *RSC Adv.* **2018**, *8* (27), 15044-15055. DOI: 10.1039/c8ra01204k.

(47) Degani, O.; Salman, H.; Gepstein, S.; Dosoretz, C. G. Synthesis and Characterization of A New Cutinase Substrate, 4-Nitrophenyl (16-Methyl Sulfone Ester) Hexadecanoate. *J. Biotechnol.* **2006**, *121* (3), 346-350. DOI: 10.1016/j.jbiotec.2005.08.011.
(48) Shu, Z. Y.; Yang, J. K.; Yan, Y. J. Purification and Characterization of a Lipase from *Aspergillus niger* F044. *Chinese J. Biotechnol.* **2007**, *23* (1), 96-100. DOI: 10.1016/s1872-2075(07)60007-7.
(49) Muhannad Massadeh, F. S., Rana Dajani, and Alaa Arafat. Purification of Lipase Enzyme Produced by *Bacillus Stearothermophilus* HU1. ICeBS Penang 2012.
(50) He, X. A Continuous Spectrophotometric Assay for the Determination of Diamondback Moth Esterase Activity. *Arch. Insect. Biochem. Physiol.* **2003**, *54* (2), 68-76. DOI: 10.1002/arch.10103.
(51) Kim, H. E.; Park, K. R. Purification and Characterization of An Esterase from *Acinetobacter lwoffii* I6C-1. *Curr. Microbiol.* **2002**, *44* (6), 401-405. DOI: 10.1007/s00284-001-0008-6.
(52) Praveen, K.; Usha, K. Y.; Viswanath, B.; Reddy, B. R. Kinetic Properties of Manganese Peroxidase from the Mushroom *Stereum ostrea* and Its Ability to Decolorize Dyes. *J. Microbiol. Biotechnol.* **2012**, *22* (11), 1540-1548. DOI: 10.4014/jmb.1112.12011.
(53) Iqbal, H. M.; Asgher, M. Characterization and Decolorization Applicability of Xerogel Matrix Immobilized Manganese Peroxidase Produced from *Trametes versicolor* IBL-04. *Protein Pept. Lett.* **2013**, *20* (5), 591-600. DOI: 10.2174/0929866511320050013.
(54) Munir, I. Z.; Dordick, J. S. Soybean Peroxidase as An Effective Bromination Catalyst. *Enzyme. Microb. Technol.* **2000**, *26* (5-6), 337-341. DOI: 10.1016/s0141-0229(99)00180-5.
(55) Mukherjee, D.; Taylor, K. E.; Biswas, N. Soybean Peroxidase-Catalyzed Oligomerization of Arylamines in Water: Optimization, Kinetics, Products and Cost. *J. Env. Chem. Eng.* **2020**, *8* (4), 103871.
(56) Mukherjee, D.; Bhattacharya, S.; Taylor, K.E.; Biswas, N. Enzymatic Treatment for Removal of Hazardous Aqueous Arylamines, 4,4′-Methylenedianiline and 4,4′-Thiodianiline. *Chemosphere* **2019**, *235*, 365–372.
(57) Cordova-Villegas, L.G.; Cordova-Villegas, A.Y.; Taylor, K.E.; Biswas, N. Response Surface Methodology for Optimization of Enzyme-Catalyzed Azo Dye Decolorization. *J. Environ. Eng.* **2019,** *145* (5), 1–10.
(58) Villegas, L. G. C. Enzymatic Treatment of Azo-dyes With Soybean Peroxidase. University of Windsor, Ontario, Canada, 2017.
(59) Roy, S. K.; Kulkarni, A. P. Isolation and Some Properties of Dioxygenase and Co-Oxidase Activities of Adult Human Liver Cytosolic Lipoxygenase. *J. Biochem. Toxicol.* **1996**, *11* (4), 161-174. DOI: 10.1002/(SICI)1522-7146(1996)11:4<161::AID-JBT1>3.0.CO;2-I.
(60) Guzik, U.; Hupert-Kocurek, K.; Sitnik, M.; Wojcieszyńska, D. High Activity Catechol 1,2-Dioxygenase from *Stenotrophomonas maltophilia* Strain KB2 as A Useful Tool in *Cis*, *C*is-Muconic Acid Production. *AvL* **2013**, *103*, 1297–1307.
(61) Shukla, S. P.; Modi, K.; Ghosh, P. K.; Devi, S. Immobilization of Horseradish Peroxidase by Entrapment in Natural Polysaccharide. *J. Appl. Poly. Sci.* **2003**, *91* (4), 2063-2071.
(62) Zhang, H.; Dierkes, R. F.; Perez-Garcia, P.; Costanzi, E.; Dittrich, J.; Cea, P. A.; Gurschke, M.; Applegate, V.; Partus, K.; Schmeisser, C.; et al. The Metagenome-Derived Esterase PET40 is Highly Promiscuous and Hydrolyses Polyethylene Terephthalate (PET). *FEBS J.* **2024**, *291* (1), 70-91. DOI: 10.1111/febs.16924.
(63) Margino, S.; Nugroho, A.J.; Asmara, W. Purification and Characterization of *Streptomyces sp.* IK Chitinase. *Indones. J. Biotechnol.* **2010**, *15* (1), 29-36.

(64) Zarei, M.; Aminzadeh, S.; Zolgharnein, H.; Safahieh, A.; Daliri, M.; Noghabi, K. A.; Ghoroghi, A.; Motallebi, A. Characterization of a Chitinase with Antifungal Activity from A Native *Serratia marcescens* B4A. *Braz J. Microbiol.* **2011**, *42* (3), 1017-1029. DOI: 10.1590/S1517-83822011000300022.
